# A Case Report of a Clinically Suspected Diagnosis of Mitochondrial Encephalomyopathy, Lactic Acidosis, and Stroke-Like Episodes (MELAS) Syndrome With Cardiac Impairment

**DOI:** 10.7759/cureus.56980

**Published:** 2024-03-26

**Authors:** Rabiu Momoh, Sam Kollamparambil

**Affiliations:** 1 Critical Care, William Harvey Hospital, Ashford, GBR

**Keywords:** hyperlactatemia, melas cardiomyopathy, heart failure, maternal inheritance, rare diseases, mitochondrial disorder, cardiomyopathy, cardiology imaging, genetic syndromes, melas

## Abstract

This case report presents a description of a hypertrophic left ventricle with reduced ejection fraction in a man in his mid-twenties with clinical, radiologic, and biochemical features of a rare syndrome called mitochondrial encephalomyopathy, lactic acidosis, and stroke-like episodes (MELAS). A literature review of this uncommon syndrome and MELAS cardiomyopathy has been conducted.

## Introduction

Mitochondrial encephalomyopathy, lactic acidosis, and stroke-like episodes (MELAS) syndrome is a rare condition with a genetic importance (with mutations affecting MT-ND1, MT-ND5, MT-TH, MT-TL1, and MT-TV genes) and a maternal inheritance pattern to it [1]. This syndrome was first described by Pavlakis et al. in 1984 [2]. It causes impaired mitochondrial function and resultant hyperlactatemia. Patients experience severe lethargy, myalgia, recurrent headaches, anorexia, vomiting, and seizures. They may experience stroke-like episodes before age 40, causing progressive brain damage and possible onset of dementia [1]. Hypertrophic cardiomyopathy preceding dilated cardiomyopathy is the natural progression of cardiac dysfunction that can affect MELAS patients [3].

MELAS syndrome is the most common mitochondrial disorder. It has a prevalence of 0.18 per 100,000 in Japan, 1.41 per 100,000 in the northeast of England, 2 per 100,000 in Sweden, 18.4 per 100,000 in Finland, and 236 per 100,000 in Australia [4].

We present the case of a male patient in his mid-twenties who presented in status epilepticus to the hospital. Upon further in-hospital assessments, a clinical diagnosis of a rare condition called MELAS syndrome with MELAS cardiomyopathy was made on the patient.

## Case presentation

A 25-year-old male patient was brought in by ambulance crew to the hospital in status epilepticus (refractory to intravenous lorazepam boluses) and was noted to have significantly aspirated. He was found in his parked work van having repeated generalized tonic-clonic seizures. He was on a sojourn by road from Holland to the UK. He needed high inspired oxygen fractions (fio2 80%) to maintain pulse oximetry saturations at 92% and had coarse crackles in the right upper lung zones on auscultation. Intravenous levetiracetam and then phenytoin were loaded as his seizures continued. He had endotracheal intubation done in the intensive care unit, and further sedation and mechanical ventilation were initiated. The patient had intravenous antimicrobials initiated (ceftriaxone and acyclovir) pending lumbar puncture and cerebrospinal fluid (CSF) analysis. Respiratory screens for COVID-19, influenza A and B, and respiratory syncytial virus were negative, and so were screens for legionella and mycoplasma. Urine pneumococcal antigen screen was also negative. Blood screens for paracetamol and salicylate toxicities were negative.

**Figure 1:**
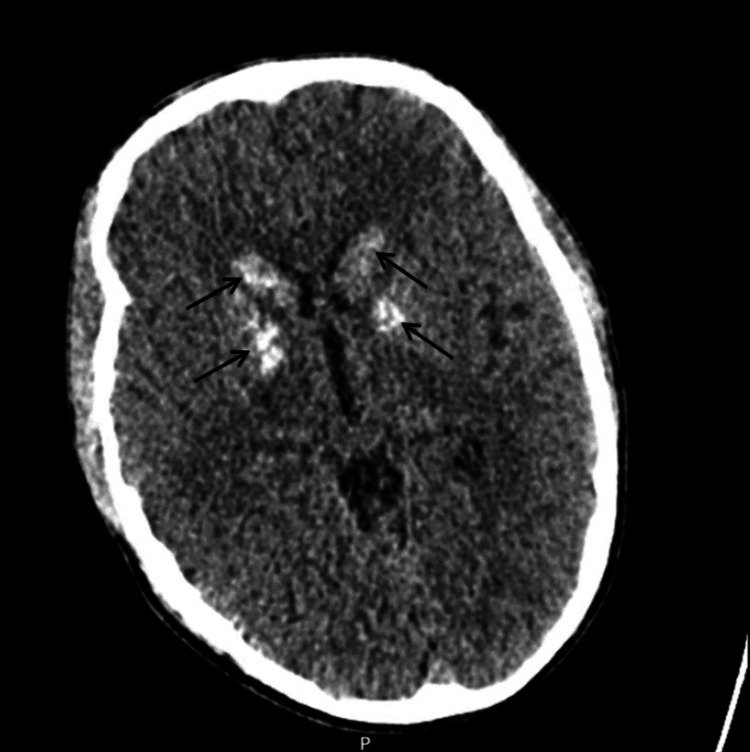
Transverse section through a non-contrast CT head study revealing hyper-attenuation in both basal ganglia (marked out with indicating arrows in the figure)

Non-contrast CT head study done revealed relatively symmetrical areas of hyper-attenuation within the basal ganglia and no other major pathology seen elsewhere in the brain (Figure 1).

The collateral history obtained revealed the patient had no recent fevers, cough, or change in normal bowel or urinary habits, nor had he been treated for any recent infections. No history of ethanol abuse or cigarette use was reported. He had a history of epilepsy that was treated with oral levetiracetam, and he had been seizure-free for five years until this current episode. The patient had a family history of MELAS syndrome and had lost a brother due to unknown complications due to this condition. He and his sister were reported as carriers for this condition (genetic study reports were not available).

No sign of head trauma was found on inspection. He was asthenic. His pupils were equal, round, and reactive to light. He had a normal tone globally, and there was no clonus. Reflexes were normal globally. His heart rate was 100 beats per minute while sedated and mechanically ventilated. Blood pressures were within normal limits. His heart sounds were S1 and S2. The patient's abdomen was noted to be mildly distended but non-tender at admission, with no palpable organomegaly. An abdominopelvic CT scan done revealed notable gastric distension (with no gastric outlet obstruction), and the rest of the unprepared bowel appeared to be of normal calibre. The liver, spleen, kidneys and pancreas were of normal review.

**Figure 2:**
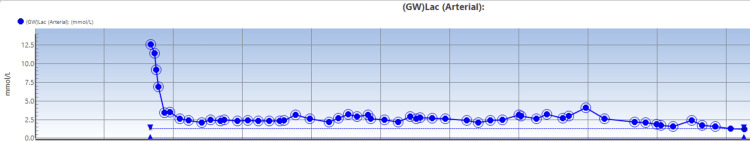
Serial blood lactate checks Spaces between the black vertical lines represent progressive days spent in the hospital from right to left. The dotted horizontal blue line represents the upper limit of normal for blood lactate

**Figure 3:**
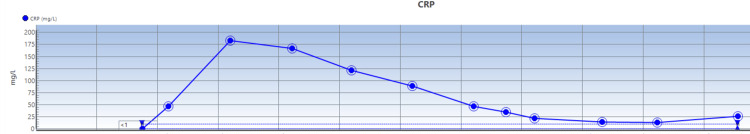
Serial CRP measurements Spaces between the black vertical lines represent progressive days spent in the hospital from right to left CRP - C-reactive protein

**Figure 4:**
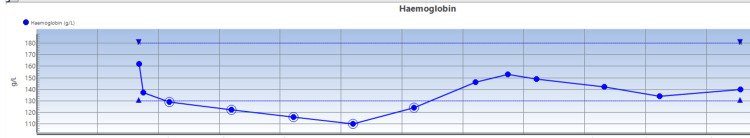
Serial haemoglobin measurements in the patient Spaces between the black vertical lines represent progressive days spent in the hospital from right to left

**Figure 5:**
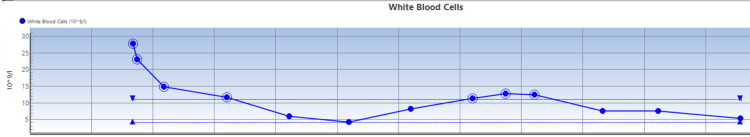
Serial blood white cell counts Spaces between the black vertical lines represent progressive days spent in the hospital from right to left

A lumbar puncture was done, and the cerebrospinal fluid (CSF) analysis can be found below in Table 1. The patient's acyclovir use was discontinued after his CSF viral screen returned negative. Blood study results done within the early hours of hospital admission are provided in Table 2. The patient's blood lactate level on the first two days of admission ranged between 3.4 and 12.5mmol/l and thereafter ranged between 1.2 and 4.1 mmol/l (ref range: 0 - 1.2 mmol/l). See trends of blood lactate (Figure 2), c-reactive protein (CRP) (Figure 3), haemoglobin (Figure 4) and white cell counts (Figure 5) below.

**Table 1:** CSF study results CSF - cerebrospinal fluid

Investigation	Result	Reference range / comment
CSF appearance	Clear and colourless	
CSF protein	0.45 g/l	0.15-0.45 g/l
CSF glucose	3.2 mmol/l	Normal
CSF lactate	4.4 mmol/l	1.1-2.4 mmol/l
CSF viral screen	Negative study	
CSF red cell count	<40/ml	
CSF culture	No bacterial growth identified after 48 hours of incubation	

**Table 2:** Blood studies

Investigation	Result	Reference range
Creatinine	80 umol/l	64-104 umol/l
Sodium	141 mmol/l	135-145 mmol/l
Potassium	4.3 mmol/l	3.5-5.5 mmol/l
Urea	6.2 mmol/l	2.5-7.7 mmol/l
Creatine kinase	584 U/l	40-320 U/l
Procalcitonin	1.2 ug/l	< 0.5 ug/l
Albumin	53 g/l	30-50 g/l
Alkaline phosphatase	122 U/l	30-130 U/l
Alanine transaminase	45 U/l	0-75 U/l
Total bilirubin	< 5 umol/l	0-29 umol/l
Estimated glomerular filtration rate	118 ml/min/1.73m^2^	>90 ml/min/1.73m^2^
C-reactive protein	<1 mg/l	0-10 mg/l
Haemoglobin	162 x10^9^/l	130-180 x 10^9^/l
White blood cells	27.7 x 10^9^/l	4-11 x 10^9^/l
Platelet	260 x 10^9^/l	150-400 x 10^9^/l
Neutrophil	22.6 x 10^9^/l	2-7.5 x 10^9^/l
Blood lactate level range (first two days of admission)	3.4-12.5mmol/l	0 - 1.2 mmol/l
Blood lactate level range (remaining eight days used on the intensive care unit)	1.2-4.1 mmol/l	0 - 1.2 mmol/l
HIV 1+2	Non-reactive	
Syphilis IgG and IgM screens	Negative	

**Figure 6:**
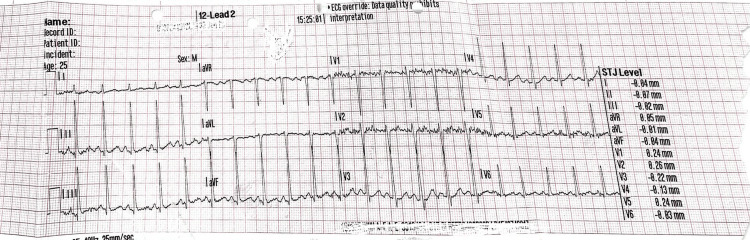
12-lead ECG done at admission in the patient revealing sinus tachycardia

**Figure 7:**
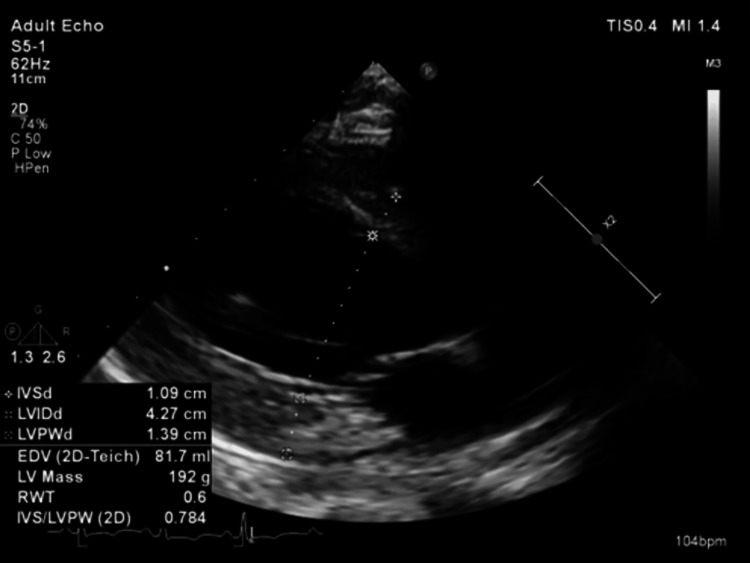
Parasternal long-axis view revealing concentric left ventricular hypertrophy with normal cavity size

**Figure 8:**
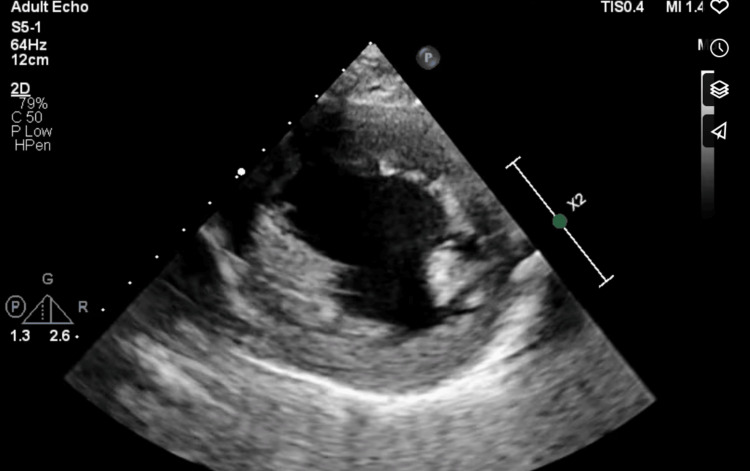
Parasternal short axis view revealing concentric left ventricular hypertrophy and trabeculation

**Figure 9:**
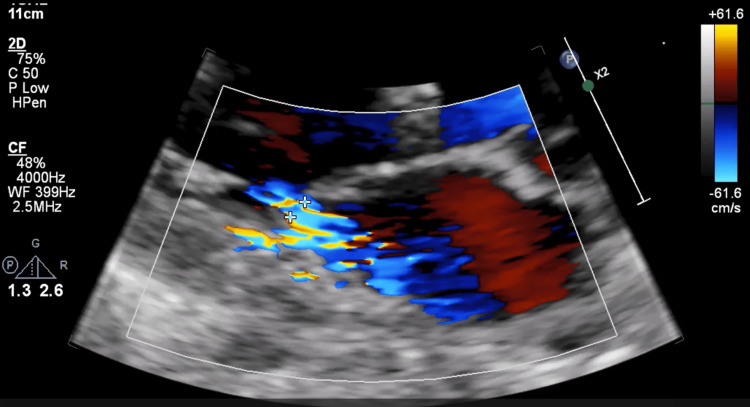
Parasternal long axis-view showing upper mild mitral regurgitation

The patient was also reported to have an unnamed heart condition. An electrocardiogram done at admission revealed sinus tachycardia, heart rate at the time of study was 120 beats per minute (Figure 6). Echocardiography done following discontinuation of mechanical ventilation revealed mild concentric left ventricular hypertrophy with normal cavity size (Figure 7, Figure 8) - evident by thickened posterior wall measured in end-diastole (1.39 cm ref: mildly abnormal (1.1-1.3 cm) moderately abnormal (1.4-1.6 cm)), thickened interventricular septum thickness measured in end-diastole (finally assessed as 1.37 cm ref. mildly abnormal (1.1-1.3 cm) moderately abnormal (1.4-1.6 cm)). He had an impaired left ventricular (LV) systolic function, with an ejection fraction (EF) of 30‐35%. Global LV hypokinesia was noted (Video 1). Left ventricular apical trabeculations were noted. The left ventricular outflow tract diameter is 1.95cm (normal should be above 2cm in males). The aortic valve appeared tricuspid. Its cusps were thin and mobile with good excursion. No aortic regurgitation was present. A degenerate posterior wall mitral valve leaflet was noted (Video 2). A mild to almost moderate mitral regurgitation was seen. The eccentric jet is posteriorly directed (Figure 9). Both atria were of normal sizes. The right ventricle was of normal size and contractility. TAPSE 19mm. Tricuspid valve leaflets were thin and mobile with good excursion. There was trivial tricuspid regurgitation. The pulmonic valve was normal in structure and function. Trivial pulmonary regurgitation was noted. He had normal aortic root size. There was no pericardial effusion. Other echocardiographic measurements are provided in Table 3.

**Video 1:** Apical 4-chamber echocardiogram view revealing left ventricular (LV) hypertrophy, impaired LV systolic function, global LV hypokinesia and degenerate posterior mitral valve leaflet.

**Video 2:** Echocardiogram apical 3-chamber view revealing a degenerate posterior mitral valve leaflet

**Table 3:** Finalized echocardiographic measurements IVSd - interventricular septum thickness; LVIDd - left ventricular internal dimension in diastole; LVPWd - left ventricular posterior wall thickness at end-diastole; LVIDs - left ventricular internal diameter at end-systole; EDV - end diastolic volume; MAPSE - mitral annular plane systolic excursion; SV - stroke volume; LVOT - left ventricular outflow tract; Med Peak E' Vel - medial mitral annular peak e’ wave velocity; Lat Peak E' Vel - lateral annular peak e‘ wave velocity; E/E' Lat - early diastolic mitral inflow velocity to early diastolic lateral mitral annulus velocity; E/E' Med - early diastolic mitral inflow velocity to early diastolic medial mitral annulus velocity; MV E/A - early diastolic flow velocity (E velocity) divided by late diastolic transmitral flow velocity; PA V2 max - Pulmonary artery maximum velocity; PA acc time - pulmonary acceleration time; PA Pr (Acc) - pulmonary pressure estimated by pulmonary acceleration time; Ao Sinus Diam - aortic sinus diameter; AV - aortic valve; VTI - velocity-time integral; AVA - aortic valve area; MV - mitral valve; max - maximum; vel - velocity; RV - right ventricle; TAPSE - tricuspid annular plane systolic excursion

Parameters	Measurements
Left ventricular size and systolic function	
IVSd	1.37cm
LVIDd	4.2cm
LVPWd	1.39cm
LVIDs	3.7cm
EDV(MOD‐sp4)	85.9 ml
ESV(MOD‐sp4)	53.5 ml
MAPSE	0.57 cm
SV(LVOT)	39.1 ml
Left ventricular diastolic function	
Med Peak E' vel	6.0 cm/sec
Lat Peak E' vel	6.9 cm/sec
E/E' Lat	8.9
E/E' Med	10.2
MV E/A	0.99
Pulmonary artery and pulmonary valve	
PA V2 Max	98.2 cm/sec
PA Acc time	0.12 sec
PA pr (Accel):	23.1 mmHg
Aorta/Aortic valve/ Left ventricular outflow tract	
Ao Sinus Diam	2.9 cm
LVOT diam	1.95 cm
Ao V2 max	95.6 cm/sec
AV Velocity ratio	0.84
LVOT max vel	80.6 cm/sec
LVOT VTI	13.0 cm
AVA(V,D)	2.5 cm²
Mitral Valve	
MV E max vel	61.3 cm/sec
MV A max vel	62.1 cm/sec
Right ventricular size and function	
RV Base	3.0 cm
TAPSE	1.93 cm

**Figure 10:**
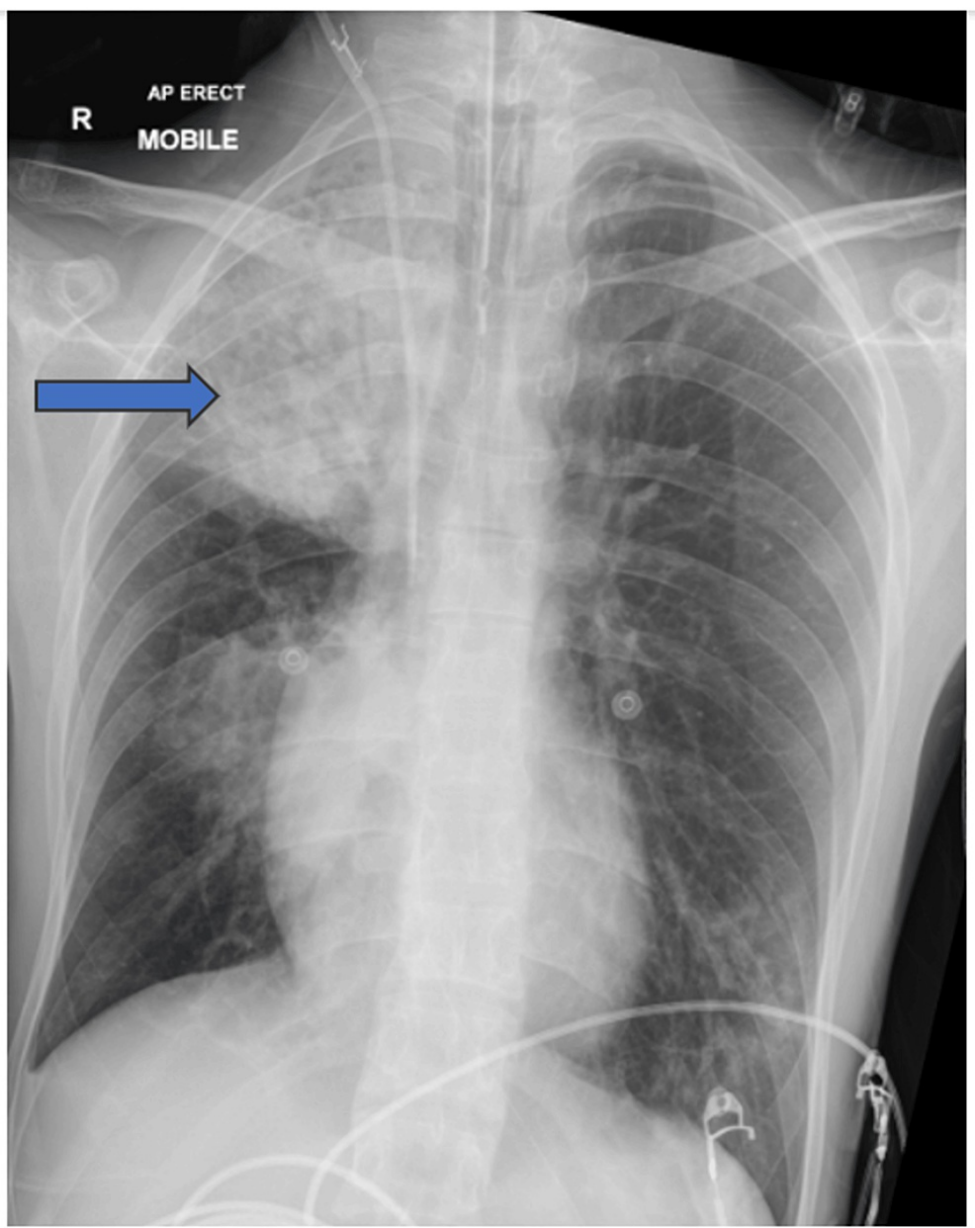
Right upper lung zone opacification from possible aspiration complicating status epilepticus in the patient

**Figure 11:**
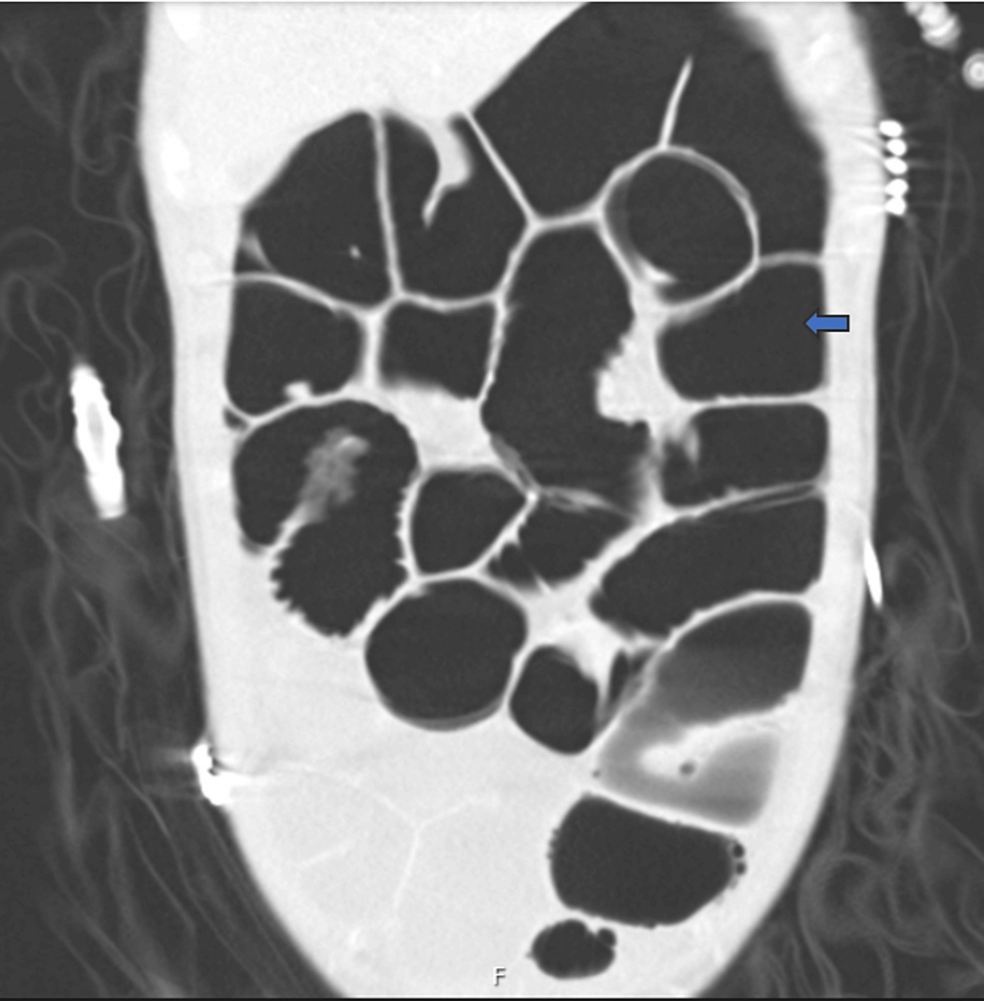
Coronal section of a CT abdomen study revealing the finding of dilated small and large bowel loops in the patient (assessed as ileus)

**Figure 12:**
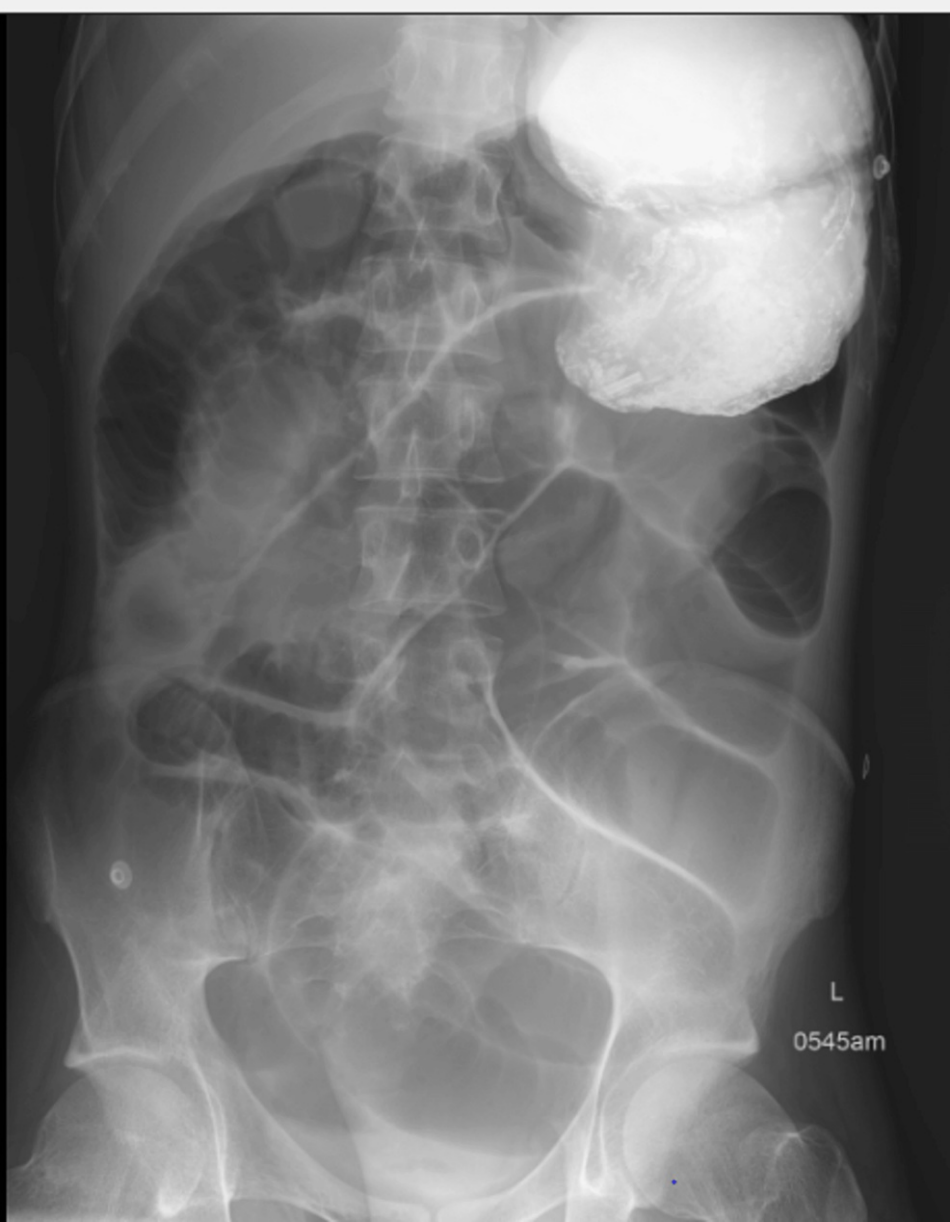
Gastrograffin X-ray of the abdomen study showing pooled contrast in the markedly dilated stomach and dilated colon loops

 A multi-disciplinary approach was used in the care of the patient in the intensive care unit. The patient had no further seizures beyond day one of hospital admission; intravenous levetiracetam was continued post-extubation. His aspiration pneumonitis (Figure 10) improved, and he was weaned off supplemental oxygen requirement. Another concurrent issue managed during his intensive care unit stay was the occurrence of ileus in this patient, as no mechanical obstruction was found on abdominal radiologic studies done (Figure 11 and Figure 12), and was managed with intravenous prokinetics. MRI Head study done on day nine of hospital admission revealed no acute parenchymal abnormality involving the cerebral or cerebellar hemispheres. There was no midline shift. No evidence of haemorrhage or infarction was seen. No mass or mass effect was noted. No restricted diffusion. The ventricles, sulci and basal cisterns were unremarkable. Normal patency of the major intra-cerebral vascular structures was seen.

Education regarding seizures and safety considerations was facilitated with the patient and his next of kin. He was stepped down to a general ward on day ten of admission and was then repatriated to his home country under the continuing care of his regular doctors on day 14. 

## Discussion

We have presented the echocardiographic findings in a young adult with clinical, laboratory and radiologic features suggesting a rare inheritable MELAS syndrome.

MELAS is a maternally inherited condition that results from mitochondrial gene mutations, causing a wide range of multi-organ affectations and mainly causing mitochondrial encephalomyopathy, lactic acidosis and stroke-like episodes. The commonest genetic mutation associated with this condition is the m.3243A>G pathogenic variant in the mitochondrial gene MT-TL1, and this accounts for 80% of MELAS occurrence [5].

A clinical assessment revealing a history of seizures and a family history suggesting MELAS, a physical feature of asthenia, CSF study finding of raised CSF lactate level, blood study revealing persistent hyperlactatemia in the patient as well as echocardiogram finding supporting possible MELAS cardiomyopathy were used to clinically diagnose MELAS syndrome in the 25-year-old male under review. An MRI head study, if done earlier in the admission, could have been a better assessment tool for the hyperattenuating lesion seen on the patient's basal ganglia revealed on CT. The patient was noted to be moving all limbs with full power prior to endotracheal intubation at admission and had no neurological deficit on assessment post-extubation. A genetic study to confirm the presence of any of the known mitochondrial DNA mutations associated with this syndrome could have been useful. Other useful investigations that were considered in this patient included muscle biopsy, electroencephalogram, and cardiac magnetic resonance imaging study (MRI). The patient was actively considered for repatriation to his home country (Holland) from his early days in the intensive care unit admission. The aforementioned investigations would have been undertaken if the patient had more time under the United Kingdom health system. Other aspects of his critical care were prioritized.

MELAS cardiomyopathy is known to complicate a third of MELAS cases. A progression from hypertrophic cardiomyopathy to eventually dilated cardiomyopathy is noted in the literature [4]. Patients with MELAS cardiomyopathy may range from being asymptomatic to manifesting features of heart failure, malignant arrhythmias, or sudden cardiac death [6]. Histological findings in MELAS cardiomyopathy include a significant increment in mitochondrial inclusions and an increased number of abnormal mitochondria. Variable sarcomere thickening and heterogeneous distribution of affected cardiomyocytes have also been noted [3].

Despite echocardiogram studies confirming a reduced left ventricular ejection fraction of 30-35%, on assessment post-extubation, the patient had neither orthopnea nor paroxysmal nocturnal dyspnea but reported easy fatiguability and dyspnea on severe exertion. He had no pedal edema and reported no chronic cough, sputum production, and did not experience palpitations. 

Tran et al. (2023) revealed that MELAS could be accompanied by diabetes mellitus (DM) in some patients - a known condition that can cause gastroparesis [7]. The patient under review however persistently had blood glucose readings within the non-diabetic limits. An HbA1C study could also be used to formally exclude diabetes. On evaluating the patient's mildly distended and non-tender abdomen at admission, a finding of gastric distension without gastric outlet obstruction was found. Horná et al. (2024) noted that gastro-intestinal manifestations of MELAS syndrome include gastroparesis, intestinal pseudo-obstruction and pancreatitis [8].

The differential diagnoses of MELAS syndrome include other mitochondrial disorders such as Kearns-Sayre syndrome, myoclonus epilepsy associated with ragged red fibres (MERRF), and Leigh syndrome [9]. The major cardiac differential diagnoses of MELAS cardiomyopathy will include Anderson-Fabry disease and other causes of hypertrophic and dilated cardiomyopathies. Anderson-Fabry may also present with stroke and other systemic features such as deafness. Cardiac MRI (multiparametric) would be useful in distinguishing between these cardiomyopathies. Other investigations like genetic or metabolic testing may take time (weeks to months) to return results. The cardiac MRI findings typical in MELAS cardiomyopathy are patchy, widespread, non-ischemic type late gadolinium-enhancing (LGE) lesions thought to arise from diffuse interstitial fibrosis, myocardial disarray, and replacement fibrosis in the setting of myocyte deaths. Mitochondrial angiopathy seen in MELAS could cause brain stroke-like episodes and, in the heart, cause myocardial inflammation and resultant fibrosis [10].

There is no curative treatment available for MELAS; it is a progressive and eventually fatal condition. Supplements such as Coenzyme Q10 (CoQ10), L-arginine, citrulline and riboflavin have been suggested to be useful in the care of MELAS patients. Other treatment approaches that have been suggested include enhancing the function of good mitochondria in them as well as avoiding mitochondrial toxins. L-arginine, in past open-label clinical trials, has been thought to be useful to prevent and to reduce the frequency of stroke-like episodes in MELAS patients. It is thought to help improve microcirculation in the body, improve endothelial function and help to reduce tissue injury from ischaemia. It is now recommended in the North American consensus guideline on mitochondrial diseases [11]. The UK consensus statement on mitochondrial disorders find the use of L-arginine in mitochondrial disease stroke-like episodes controversial and, as a result, do not recommend its use during those stroke-like episodes [12]. Other emerging treatment options being explored in mitochondrial disorders include exercise training, mitochondrial donation, hypoxic therapy, device use, tissue replacement gene therapy, and mitochondrial base editing [11]. The patient under review received dietetic input and received a daily multivitamin formulation (containing (thiamine (B1), riboflavin (B2), pyridoxine (B6) and nicotinamide) and a trace elements formulation (containing selenium, zinc, copper, iron, manganese, fluoride, molybdenum, chromium, and iodine).

In a systematic review of 21 studies and 825 mitochondrial disorder cases, Qadir et al. (2019) found more severe cardiac findings in patients with MELAS and MERRF syndrome than in other mitochondrial disorders. They suggested, because of their study, that more intense cardiac screening for patients with the above syndromes [13]. Brambilla et al. (2019) revealed that cardiac impairment occurring in the presence of other extra-cardiac MELAS features is a notable red flag in this syndrome. They also stated that cardiac involvement in paediatric MELAS patients is a marker of systemic disease severity in these patients [14]. Niedermayr (2018) suggested mitochondrial mutation screening should be done for patients with unexplained cardiac features occurring in the context of deafness, short stature and learning disabilities [15].

Cosma et al. (2023) published the case of a 55-year-old male patient with MELAS who had no other cardiovascular risk factors and had presented with non-ST-segment elevation myocardial infarction (NSTEMI). He underwent a coronary angioplasty to the posterior descending artery and the left anterior descending artery. They questioned whether there was any association between MELAS and atherosclerosis [16]. Seitun et al. described the finding of non-calcified atherosclerotic plaques with no significant obstructive coronary stenosis in a 49-year-old male MELAS patient who had presented with severe chest pain and dyspnea. Their patient received an implantable cardioverter defibrillator (ICD) as primary prophylaxis for sudden cardiac death (SCD) due to the strong family history (two maternal relatives who had had SCD) and the presence of myocardial fibrosis on cardiac MRI done on the patient [17].

Reid et al. (2021) described the cardiac findings of concentric left ventricular hypertrophy, a left ventricular ejection fraction of 40% and grade II diastolic dysfunction in a 38-year-old male with known sensorineural deafness, type 1 diabetes mellitus, who presented to a hospital's emergency section with headaches, personality change, ataxia, and facial asymmetry. Further investigations revealed MRI head findings of the right parietal lobe, occipital lobe, and temporal lobe cortical swellings, with areas of cortical diffusion restriction, basal ganglia calcification, and diffuse cerebral atrophy. Hyperlactatemia was also noted. A genetic study in their patient yielded the commonest mutation associated with MELAS: m.3243A > G mutation in the mitochondrial tRNALeu (UUR) (MTTL1) gene [10]. The case report shared by Reid et al. shares similarities with our index case report in terms of cardiac findings, findings on the brain, and hyperlactatemia.

Guideline-directed treatment of heart failure should be done for patients with MELAS cardiomyopathy. The role of cardiac device therapies could be considered in these patients. Thoughtful consideration should be given to the use of routine cardiology medications in MELAS patients. For instance, acetylsalicylic acid (aspirin) inhibits the respiratory electron transport chain (a mitochondrial pathway for ATP production). Beta-blocker medications inhibit ATPase, and statins reduce coenzyme Q10. Heart transplants can be considered for patients with MELAS cardiomyopathy while bearing an awareness of the impact of the extra-cardiac MELAS disease process in mind. Literature exploring the impact of immunosuppression on the MELAS disease process are limited. Genetic counselling in MELAS syndrome can be complicated by the variable phenotypic manifestations of MELAS phenotypes and the challenge of predicting mutant levels and expression [18].

In a retrospective study reviewing the database of 1330 children with cardiomyopathy who underwent heart transplants, divided into two groups (those with mitochondrial disorder (47) and those without), Weiner et al. revealed that the four-year median survival rate of these two patient groups was similar. They noted that those with mitochondrial disorders were at a higher risk of longer mechanical ventilation, longer intensive care unit and hospital stays, and had a higher risk of having a stroke post-transplantation. They concluded that the presence of mitochondrial disease should not be an absolute contra-indication to undertaking a heart transplant procedure [19].

Di Toro et al. (2022) reported that the high risk of strokes, presence of chronic kidney disease and wasting myopathy were factors that can mitigate against undertaking heart transplants in adult MELAS patients with MELAS cardiomyopathy. They noted these findings in a 23-year institutional review of MELAS cardiomyopathy at a referral heart transplant centre in Italy [20]. Kofler et al. (2017) reported the successful conduct of a combined heart-and-kidney transplant in a 39-year-old female with MELAS complicated by end-stage heart and kidney failure in Austria. The patient required immunosuppression post-transplantation (initially anti-thymocyte globulin and then tacrolimus, mycophenolate and prednisolone). They reported no evidence of rejection or graft vasculopathy at a follow-up assessment thirty-one months post-transplantation [21].

## Conclusions

MELAS cardiomyopathy accounts for a heightened morbidity and mortality risk in MELAS patients. Further research efforts into MELAS and MELAS cardiomyopathy should be done toward improving the quality of life and life expectancy for patients with these conditions. Multidisciplinary management (including inputs from geneticists, cardiologists, neurologists, family physicians, specialist nurses, physiotherapists, occupational therapists, and dieticians) is essential for patients with this rare syndrome and for those with ensuing MELAS cardiomyopathy complications. The role of genetic counselling is essential in this rare condition with a maternal inheritance pattern. 
